# Supplementary material for: Revisions to the Gliophorus irrigatus complex (Agaricales, Hygrophoraceae, Gliophorus, section Unguinosae) including four new species, one new combination and comparisons of basidiome vs. eDNA distributions
Source: MycoKeys. 2026 Jan 29;127:307–41. doi: 10.3897/mycokeys.127.174823 (PMC12877779; doi:10.3897/mycokeys.127.174823)
Supplement: Supplementary material 3 — Phylogenetic reconstruction of the Gliophorus sect. Unguinosae (ML tree) focused on ITS1 sequences, with Gliophorus sciophanus as outgroup [file mycokeys-127-307-s003.pdf]

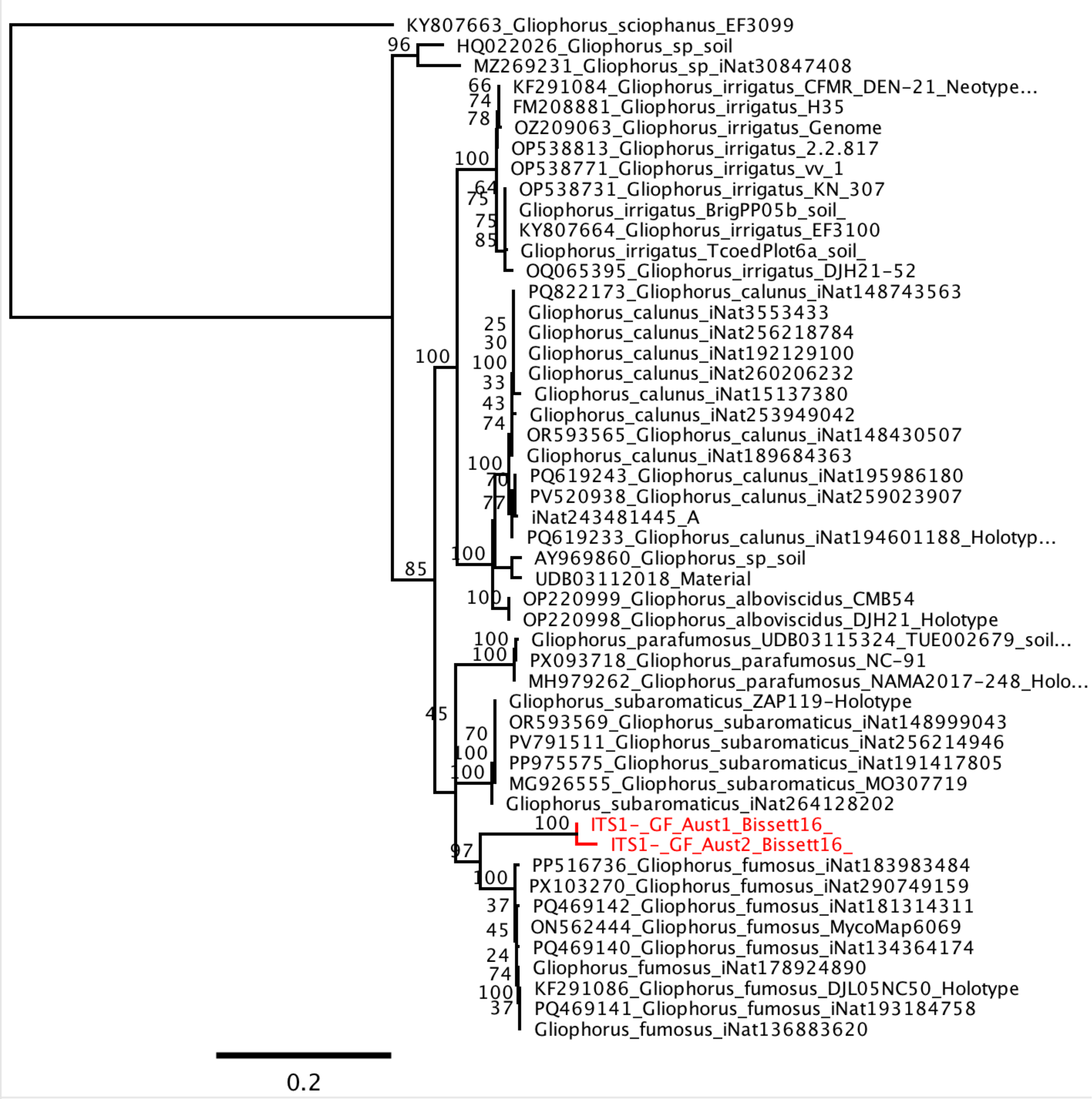

Suppdata 3. Phylogenetic reconstruction of the *Gliophorus* Sect. Unguinosae (ML tree) focused on ITS1 sequences, with *Gliophorus sciophanus* as outgroup. Numbers at salient nodes indicate % ultrafast bootstrap support (3000 replicates). The clade shown in red, adjacent to *G. fumosus*, is represented by two soil eDNA sequences from Australia (as detailed in Suppdata 1).
